# Liver myofibroblasts up-regulate monocyte CD163 expression via PGE2 during hepatitis B induced liver failure

**DOI:** 10.1186/1479-5876-12-60

**Published:** 2014-03-06

**Authors:** Min Zhang, Yinong Ye, Fenglan Wang, Jianyun Zhu, Qiyi Zhao, Yubao Zheng, Yurong Gu, Chan Xie, Zhanlian Huang, Qiang Tai, Yutian Chong, Zhiliang Gao

**Affiliations:** 1 Department of Infectious Diseases, The Third Affiliated Hospital of Sun Yat-sen University, No 600 Tianhe Road, Guangzhou 510630, Guangdong Province, People’s Republic of China; 2 Department of Infectious Diseases, The First Affiliated Hospital of Medical College of Xi’an Jiaotong University, Xi’an, People’s Republic of China; 3 Department of Hepatic Surgury, The First Affiliated Hospital of Sun Yat-sen University, Guangzhou, People’s Republic of China

**Keywords:** Monocyte activation, CD163 expression, Liver failure, Liver myofibroblasts, Hepatitis B virus

## Abstract

**Background:**

Although patients with liver failure exhibit a generalized inflammatory-imbalance status, substantial evidence indicates that this immunosuppressive or anti-inflammatory state may be deleterious. Increased expression of CD163 (known to be involved in several anti-inflammatory functions of the immune system) in patients with liver failure is significantly correlated with a fatal outcome. However, little is known of the regulatory mechanisms that influence the expression of CD163.

**Methods:**

We assessed the expression of CD163 on monocytes from both circulating cells and the liver tissues of patients with hepatitis B induced liver failure using flow cytometry and isolated the myofibroblasts from diseased livers. The ability of human liver myofibroblasts to regulate CD163 expression on monocytes was studied in vitro.

**Results:**

We showed that CD163^+^ monocytes were enriched primarily in diseased livers and that they were associated with liver myofibroblasts in the same area. Accordingly, liver myofibroblasts were significantly superior to normal skin fibroblasts in inducing the expression of CD163 on monocytes in vitro. Moreover, we found that liver myofibroblasts triggered the activation of monocytes by secreting PGE2. Inhibition of PGE2 production in liver myofibroblasts using NS-398 markedly reduced CD163 expression in vitro.

**Conclusion:**

These results suggest that liver myofibroblasts play a direct role in regulating the expression of CD163 on monocytes in human liver tissues and thereby may regulate monocyte function during hepatitis B induced liver failure.

## Background

Liver failure, whether liver insufficiency in chronic disease or occurring in acute state, remains a condition with a poor prognosis and high mortality [1]. Hepatitis B virus (HBV) induced liver failure is the most common severe disease requiring immediate hospitalization in China [2]. Monocytes and other cells of the immune system are involved in the pathogenesis of liver failure, as reflected by the activation of both pro- and anti-inflammatory cascades of the innate immune system [3]. Several investigations have suggested that changes in inflammatory balance may be important for disease course and that a shift towards an immunosuppressive or anti-inflammatory state may be deleterious [4-6].

CD163 is a member of the scavenger-receptor cysteine-rich (SRCR) protein superfamily. It is expressed strictly on cells of the monocyte/macrophage lineage and is a multifunctional molecule that fulfills essential homeostatic functions in diverse biological processes [7]. It has been suggested that CD163-positive monocytes/macrophages play a role in the resolution of inflammation because they are found in high numbers in inflamed tissue [8]. Consistent with a role in the late inflammatory response, the expression of CD163 is up-regulated in patients with liver failure. It is significantly correlated with a fatal outcome and may be used as a parameter to determine liver disease prognosis [5]. However, little is currently known of the regulatory mechanisms that influence the expression of CD163 on monocytes in liver tissues of hepatitis patients.

Prostaglandin E2 (PGE2) is a bioactive eicosanoid that regulates a variety of both innate and adaptive immune responses via four distinct G-protein-coupled receptors (EP1-4) that each show differential signaling activities and unique expression patterns in different cell types [9]. PGE2 has long been recognized as an important mediator of inflammation, and it modulates a variety of physiological processes including immune modulation function of antigen-presenting cell (APC) [10], and the production of inflammatory cytokines of macrophages [11]. Monocytes are attracted by inflammation and differentiate into a variety of subtypes depending on local mediators [12,13]. It has been reported that the monocytes are polarized to CD163^+^ macrophages in the presence of PGE2 [14].

Liver myofibroblasts (LMFs), which are principally derived from hepatic activated stellate cells (HSCs) [15], can remodel the liver stroma in response to injury. Following hepatic injury, HSCs transdifferentiate into myofibroblast-like cells (known as LMFs), which display fibrogenic and contractile properties [16]. Thus, LMFs are believed to be a major fibrogenic hepatic cell type [17]. LMFs are known to also secrete immunomodulatory compounds [18], but their effect on monocytes remains unknown.

## Methods

Human tissues were obtained from patients attending Sun Yat-sen University-affiliated hospitals. Blood were from 20 patients with hepatitis B induced liver failure (Additional file 1: Table S1) and 20 healthy individuals as controls; diseased liver tissues were from 4 patients undergoing transplantation for hepatitis B induced liver failure (Additional file 1: Table S1); healthy livers were from 3 patients undergoing surgery for hepatic hemangioma; normal skin fibroblasts were obtained from 3 patients undergoing circumcision. All the samples were anonymously coded in accordance with the local ethical guidelines, as stipulated by the Declaration of Helsinki. Written informed consent was obtained from the patients, and the protocol was approved by the Review Board of Sun Yat-sen University.

### Tissue immunohistochemistry and immunofluorescence

Paraffin-embedded and formalin-fixed samples (from 4 patients undergoing transplantation due to hepatitis B induced liver failure as showed in Additional file 1: Table S1, and 3 patients undergoing surgery due to hepatic hemangioma as healthy liver controls) were cut into 5-μm sections and were then processed for immunohistochemistry. After incubating with an antibody targeted against human CD163 (DakoCytomation, Glostrup, Denmark), adjacent sections were stained with either diaminobenzidine or 3-amino-9-ethylcarbazole using the Envision System (DakoCytomation, Glostrup, Denmark). For immunofluorescence analysis, tissues were stained using polyclonal mouse anti–human CD163 (DakoCytomation, Glostrup, Denmark) and rabbit anti–human alpha-smooth muscle actin (α-SMA, Abcam, Cambridge, MA, USA) or polyclonal mouse anti–human cyclooxygenase (COX)2 (Abcam, Cambridge, MA, USA) and rabbit anti–human fibroblast activation protein (FAP, Abcam, Cambridge, MA, USA), followed by Alexa Fluor 488– or 568–conjugated goat anti–mouse IgG and Alexa Fluor 568– or 488–conjugated goat anti–rabbit IgG (Invitrogen, Grand Island, NY, USA). Positive cells were quantified using ImagePro Plus software (Media Cybernetics) and detected using confocal microscopy (Leica, Germany).

### Isolation of LMFs

Briefly, 50 grams of liver tissue or 20 grams of foreskin sample was diced and digested using type-I collagenase (100 U/mL; GIBCO, USA) and hyaluronidase (125 U/mL; Sigma-Aldrich, St. Louis, MO) followed by mechanical homogenization in a Stomacher 60 Circulator (Seward, NY, USA). The cell suspensions derived from liver specimens were cultured in DMEM medium plus 10% FBS (GIBCO, USA). LMFs passaged for up to 3–8 passage doublings were used for experiments to minimize clonal selection and culture stress, which could occur during extended tissue culture.

### Immunofluorescent staining of LMFs

LMFs cultured on collagen-coated coverslips were fixed using 1:1 acetone/methanol (10 minutes), rinsed and prewetted with phosphate-buffered saline (PBS) containing 10% fetal calf serum (FCS, HyClone Laboratories, Logan, UT, USA) and 0.1% sodium azide and were then stained using antibodies (Abs) targeted against FAP, α-SMA or immunoglobulin (IgG) controls (Abcam, Cambridge, MA, USA) in Tris-buffered saline, pH 7.4, for 60 minutes. The cells were washed and incubated for 20 minutes with isotype-specific goat anti-mouse or goat anti-rabbit fluorescein-isothiocyanate-labeled antibodies, and the nuclei were counterstained using 4′,6′-diamidino-2-phenylindole hydrochloride (Sigma Aldrich, St. Louis, MO, USA). The cells were viewed and assessed using a fluorescence microscope (Leica, Germany), and the images were analyzed using Leica Application suite software (version 4.0).

### Isolation of monocytes

Peripheral blood mononuclear cells (PBMCs) were isolated from the buffy coats derived from the blood of healthy donors using ficoll density gradients. The monocytes were selected from the PBMCs using anti-CD14-labeled magnetic beads (Miltenyi Biotec, Bergisch Gladbach, Germany). Fresh tissue monocytes were obtained according described methods [19]. Briefly, the surgical liver samples (n = 7, 4 patients undergoing transplantation due to hepatitis B induced liver failure as showed in Additional file 1: Table S1, and 3 patients undergoing surgery due to hepatic hemangioma as healthy liver controls) were cut into small pieces and digested in RPMI 1640 medium supplemented with 0.05% collagenase IV (Sigma Aldrich, St. Louis, MO, USA), 0.002% DNase I (Roche, Indianapolis, Indiana, USA) and 20% FCS at 37°C for 20 minutes. The dissociated cells were filtered through a 150-μm mesh and separated by ficoll centrifugation. The mononuclear cells were washed and resuspended in medium supplemented with 1% heat-inactivated FCS for flow cytometry analyses.

### Coculture of monocytes with LMFs or normal skin fibroblasts

The monocytes were cultured in DMEM containing 10% FCS in 48-well flat-bottomed microtiter plates (2.5 × 10^5^ cells per well) either in the absence or presence of LMFs or normal skin fibroblasts (monocyte/LMF or normal skin fibroblast ratio: 5/1). At the indicated time intervals, the monocytes were harvested, counted and analyzed. When indicated, 5 μΜ NS-398 (Cayman Chemical, Ann Arbor, MI, USA) was added at the onset of coculture.

### Enzyme-linked immunosorbent assay analysis of cell supernatants

Supernatants were generated by seeding 5 × 10^4^ cells per well into 48-well plates in 500 μL of DMEM/1% bovine serum albumin (BSA) containing 2 mmol/L L-glutamine, 60 μg/mL benzylpenicillin and 100 μg/mL streptomycin (all purchased from Sigma Aldrich, St. Louis, MO, USA). The conditioned supernatants were examined using a PGE2 enzyme-linked immunosorbent assay (ELISA, R&D Systems, Abingdon, UK).

### Western blotting

Proteins from monocytes and LMFs/skin fibroblasts were extracted as previously described [20]. Equal amounts of proteins were separated by 10% SDS-PAGE and immunoblotted using antibodies targeted against COX2 (Abcam, Cambridge, MA, USA) and GAPDH (Santa Cruz Biotechnology, Santa Cruz, CA, USA).

### Flow cytometry

The peripheral blood monocytes, liver monocytes and LMFs were stained using fluorochrome-conjugated antibodies targeted against CD14, CD163, CD31, CD45, CD34 or control antibodies (eBioscience, San Diego, CA, USA) according to the manufacturer’s instructions. The cells were subsequently analyzed using multicolor flow cytometry (FACS Vantage-SE, BD Immunocytometry Systems, San Diego, CA, USA).

### Statistical analysis

The results are expressed as the means ± SEM. Normality was tested using the Shapiro–Wilk test, and the normally distributed data were compared using paired t-tests for related samples, analysis of variance, or independent t-tests. Non-normally distributed data were compared using the Wilcoxon signed-ranks test for related samples or the Mann–Whitney U-test for independent samples. SPSS statistical software (version 13.0) was used for all of the statistical analyses. Unless otherwise specified, all of the data were analyzed using two-tailed tests, and P < 0.05 was considered to be statistically significant.

## Results

### CD163^+^ monocytes are greatly increased both in the circulation and in liver tissues of patients with hepatitis B induced liver failure

**Figure 1:**
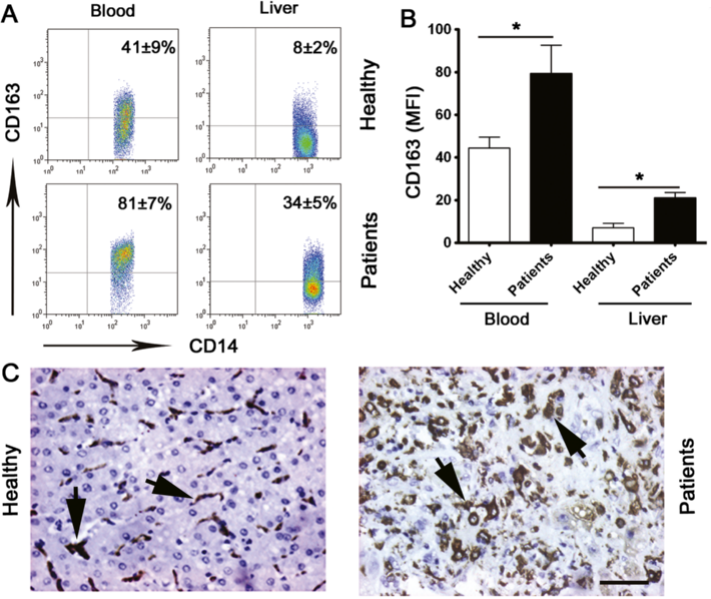
**CD163**^**+ **^**monocytes are highly increased in both the circulation and the liver tissues of patients with hepatitis B induced liver failure. (A, B)** Flow cytometry analysis of CD163 expression on freshly isolated monocytes from peripheral blood and tissues. The samples were collected from patients and donors as follows: blood from 20 healthy individuals and 20 patients with hepatitis B induced liver failure; and healthy livers from 3 patients with hepatic hemangioma, and diseased livers from 4 patients with hepatitis B induced liver failure. The percentage of CD14^+^ monocytes expressing CD163 **(A)** and the mean fluorescence intensity (MFI) of CD163 expression on CD14^+^ monocytes **(B)** are shown. The data shown in **A** are representative dot plots of individuals from more than three independent experiments; **B** shows the statistical analysis of these samples. The results are expressed as the means ± SEM. Significant differences in comparison with healthy controls are indicated (*P < 0.05). **(C)** Adjacent sections of paraffin-embedded liver samples of both healthy liver and diseased liver stained with a CD163-targeted antibody, and the typical positive cells are shown as pointed by arrows. 1 out of 15 representative micrographs is shown. Bar, 200 μm.

Clinical evidence from liver-failure patients suggests a close association between the level of CD163 expression in serum and a fatal disease outcome [5]. To examine whether CD163 is also enriched in diseased liver tissues, we utilized flow cytometry to study the surface expression of this protein on infiltrating monocytes that were freshly isolated from diseased or healthy human liver tissues and on circulating monocytes from patients with liver failure or healthy donors. Specifically, in all of the samples analyzed, CD14^high^ cells were considered as monocytes. CD163 was highly expressed on most of the circulating monocytes in patients (81 ± 7%) versus healthy controls (41 ± 9%). Compared with healthy-liver infiltrating monocytes, the monocytes isolated from the diseased liver tissues comprised a significantly greater proportion of CD163^+^CD14^high^ cells (34 ± 5% versus 8 ± 2% of healthy controls), and they expressed significantly higher levels of CD163 (P < 0.05; Figure 1A and B). Immunohistochemical analysis of hepatic monocytes confirmed that CD163 was significantly increased in the liver tissue of patients with hepatitis. As shown in Figure 1C, not only the percentage of CD163^+^ cells was increased in diseased liver infiltrating monocytes but also the intensity of CD163 expression was up-regulated.

### CD163^+^ monocytes are associated with LMFs in situ

**Figure 2:**
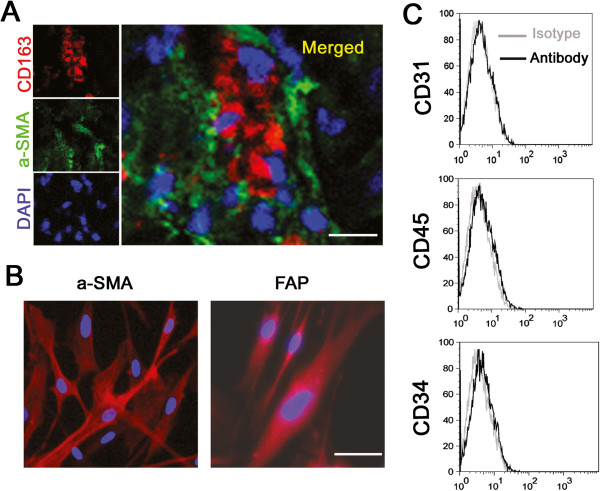
**LMFs are in close vicinity to CD163**^**+ **^**monocytes in situ, and the phenotypic characterization of LMFs isolated from human liver tissue. (A)** Analysis of LMF (α-SMA^+^) and monocyte (CD163^+^) distribution in diseased liver samples using confocal microscopy. The micrographs represent the association of LMFs and CD163^+^ monocytes; 1 out of 10 representative micrographs is shown. **(B)** Immunofluorescent in vitro anti-α-SMA and FAP staining of LMFs isolated from a representative sample of diseased livers. **(C)** The surface markers on LMFs cultured for 3–5 population doublings were determined by flow cytometry. The purity of the LMFs was confirmed using endothelial, epithelial and hematopoietic markers (CD31, CD45 and CD34). Bar, 50 μm.

We subsequently examined the distribution of CD163^+^ monocytes in diseased liver samples. Using confocal microscopy, we confirmed that CD163^+^ monocytes were associated with LMFs that expressed high levels of the α-SMA protein (Figure 2A). To further determine whether LMFs regulate the expression of CD163 on monocytes in the inflamed liver environment, we examined the in vitro differentiation of LMFs. As shown in Figure 2B, the LMFs isolated from liver tissues were characterized by high expression of α-SMA and FAP, which are expressed mainly on fibroblasts. Additionally, staining for CD31, CD45 and CD34 was used to exclude the contamination with endothelial, epithelial and hematopoietic cells. Importantly, we did not observe any differences in the phenotype of the LMF subsets between the different patients with hepatitis B induced liver failure (data not shown), suggesting that the qualitative changes in the LMF compartment represent a somewhat uniform response during fibrogenesis.

### LMFs directly up-regulate CD163 expression on monocytes in vitro

The role of LMFs in regulating monocyte CD163 expression has not been determined. Using isolated LMFs in vitro, other investigators have studied the recruitment effects of LMFs on lymphocyte infiltration and positioning [18]. Here, we examined the alteration of the activated monocyte marker CD163 in the presence or absence of LMFs. Because no LMFs exist in healthy livers and because it is difficult to use primary stable hepatic stellate cells as controls for fast activation in in vitro culture conditions, we used normal skin fibroblasts as controls, as used by other investigators [18].

**Figure 3:**
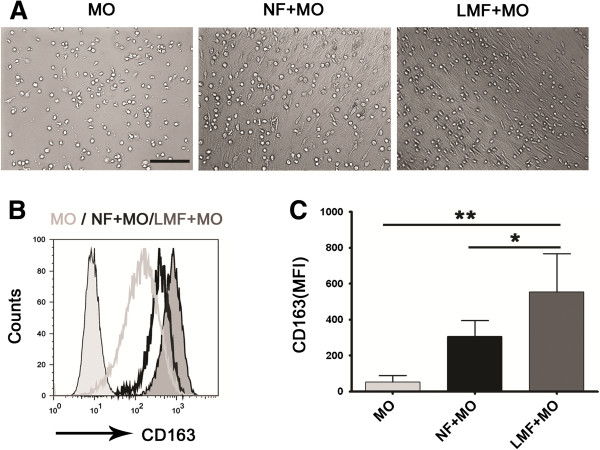
**LMFs can up-regulate CD163 expression on monocytes. (A, B)** Monocytes were left untreated (MO) or were cocultured with normal skin fibroblasts (NF + MO) or LMFs (LMF + MO) for 6 days. Lightgray filled profile in **B** show the isotype control. The histograms are representative of six separate experiments. **(C)** Statistical analysis of the MFI of CD163 expression on monocytes following 6 days in coculture. The values shown in **C** represent the means ± SEM of six separate experiments. **(P < 0.01) and *(P < 0.05) indicate significant differences compared with the untreated monocytes or the monocytes cocultured with normal skin fibroblasts **(C)**. Bar, 200 μm.

Monocytes, freshly isolated from unrelated healthy donors, were cocultured with different LMFs or normal skin fibroblasts for 6 days (Figure 3A). The expression of CD163 on monocytes was then analyzed using flow cytometry. Monocytes exposed to LMFs demonstrated enhanced CD163 expression compared with monocytes alone and those from normal skin fibroblast controls at 6 days (Figure 3B and C). Of note, normal skin fibroblasts could also partially affect CD163 expression on monocytes. However, the modulation effect of LMFs was more robust than that of normal skin fibroblasts (Figure 3B and C). Therefore, different mechanisms may underlie the activation of monocytes exposed to fibroblasts of different origins.

**Figure 4:**
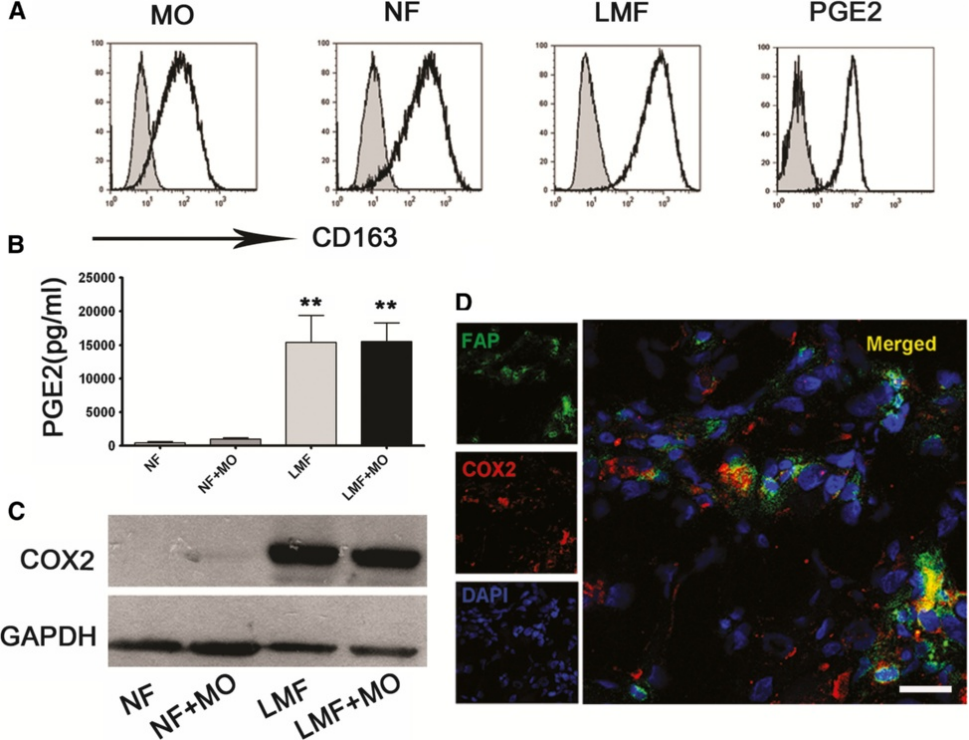
**LMFs up-regulate CD163 expression on monocytes via PGE2. (A, B)** Monocytes were left untreated (MO) or were cultured with the indicated supernatants (NF, supernatants from normal skin fibroblasts; LMF, supernatants from LMFs) or PGE2 (25 ng/mL) for 6 days. CD163 expression on monocytes was determined using flow cytometry, and the production of PGE2 in supernatants was assessed using an ELISA. The histograms in **A** are representative of five separate experiments. Gray filled profile show the isotype control. Values shown in **B** represent the means ± SEM of five separate experiments. **(P < 0.01) indicates a significant difference compared with untreated monocytes or normal skin fibroblast/monocyte cocultures. **(C)** Expression of COX2 by normal skin fibroblasts (NF), LMFs (LMF) or cocultures of monocytes (NF + MO or LMF + MO). **(D)** Analysis of the expression of COX2 on LMFs (FAP^+^) in diseased liver samples using confocal microscopy. The micrographs show that LMFs express COX2 in situ; 1 out of 10 representative micrographs is shown. Bar, 50 μm.

To obtain further insight on how LMFs modulated CD163 expression on monocytes, a protocol was designed to determine whether the enhancing effect was due to a diffusible factor or if direct cell-to-cell interaction was required. Purified monocytes were cultured in conditioned medium from normal skin fibroblasts or in conditioned medium from LMFs. We discovered that the supernatant from LMFs also effectively induced an increased expression of CD163 (Figure 4A), suggesting certain diffusible factors were secreted that modulated the monocytes.

Altogether, these results suggest that LMFs play a critical role in maintaining the up-regulation of CD163 on monocytes in the inflamed liver environment in humans.

### LMFs up-regulate CD163 expression on monocytes via PGE2

The aforementioned observations indicate that LMFs may supply locally acting paracrine cues that induce CD163 expression on monocytes within the liver inflamed environment. It is believed that monocytes are polarized to CD163^+^ differentiation in the presence of PGE2 [14], and most stromal cells demonstrate the presence of large quantities of PGE2 [21]. We conjectured that LMFs may up-regulate CD163 expression of monocytes via PGE2. To investigate this theory, in vitro cultures of LMFs/normal skin fibroblasts alone or with monocytes were established and their conditioned media were screened for the levels of PGE2 using an ELISA (Figure 4B). Notably, the levels of PGE2 in LMFs cultures alone or with monocytes were higher than those produced by cultures containing normal skin fibroblasts (Figure 4B). Consistent with this result, the expression of COX2 (an inducible enzyme involved in the production of PGE2) was highly enriched in LMFs (Figure 4C). This difference may explain why monocytes show the increased expression of CD163 when cocultured with LMFs. In support of this conclusion, the coexistence of COX2 and FAP (a protein representative of LMFs) in situ was further confirmed by confocal microscopic analysis of frozen, diseased liver tissues (Figure 4D).

We next determined whether PGE2 secreted from LMFs was sufficient to up-regulate the expression of CD163 on monocytes. To assess whether PGE2 acted directly on monocytes, we analyzed its effect using monocytes that had been cultured for 6 days in the presence of 25 ng/mL PGE2. The results showed that monocytes cultured in the presence of PGE2 displayed a marked increase in the surface expression density of CD163 (Figure 4A). Remarkably, this change occurred at concentrations that were detectable in our monocyte/LMF cocultures.

**Figure 5:**
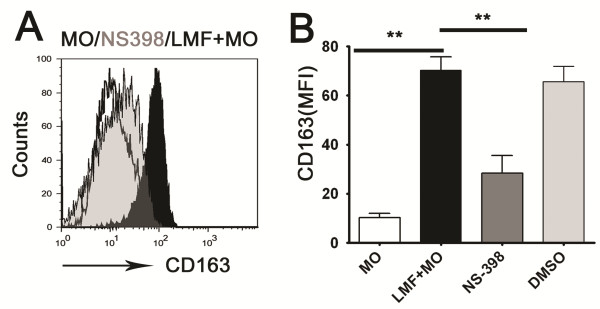
**Abolishing PGE2 production in LMFs using NS-398 can affect CD163 expression on monocytes. (A, B)** Monocytes were left untreated (MO, open profile with black solid line) or were pretreated with LMFs (LMF+MO, black filled profile) and then incubated with NS-398 (NS398, Gray filled profile) or DMSO (as control) at the indicated concentrations (5 μM). The histograms in **A** are representative of five separate experiments. Values shown in **B** represent the means ± SEM of five separate experiments. **(P < 0.01) indicates a significant difference compared with untreated monocytes or LMF-exposed monocytes incubated with NS-398.

To examine whether PGE2 was required to mediate the effects of LMF regulation of CD163 expression on monocytes, we used 5 μΜ NS-398 (a selective inhibitor of cyclooxygenase-2) to abolish the effects of PGE2. As expected, NS-398 effectively inhibited the up-regulation of CD163 (Figure 5A and B).

## Discussion

This study reveals a previously uncharacterized regulation of LMFs on monocytes in vitro, which is conserved during the development of hepatitis B induced liver failure. Whereas previous studies have shown that the monocytes play a crucial role in hepatic fibrogenesis by enhancing hepatic stellate cell activation [22,23], our data demonstrate that LMFs from inflamed liver modulate CD163 expression on monocytes. An intriguing finding of our work is that NS-398 treatment significantly suppresses CD163 expression, supporting a model in which PGE2 is required to mediate monocyte activation in vitro. The mechanisms of LMF regulation of monocytes not only highlights a novel link between inflammation and fibrosis in the liver but also expands potential therapeutic interventions to enable modulation of the complex signaling networks in the treatment of liver failure.

Monocytes/macrophages are central to the pathogenesis of liver failure because they secrete large quantities of both pro- and anti-inflammatory cytokines and are responsible for antigen presentation via their surface expression of HLA class II molecules [4,6]. CD163 is a monocyte/macrophage lineage-specific scavenger receptor and is known to be involved in several anti-inflammatory functions of the immune system [24]. The receptor is significantly up-regulated on liver macrophages during hepatitis [25]. Highly elevated levels of a circulating form of the receptor have recently been described in sera from patients with liver failure [5]. We discovered that CD163 is highly expressed on monocytes in liver tissues and in the circulation of patients with hepatitis B induced liver failure, indicating that CD163^+^ monocytes could be involved in the pathogenesis and clinical course of liver failure.

CD163 has been proposed as a protective system, and it is coherently upregulated during inflammatory conditions to enhance hemoglobin clearance and heme degradation [26]. In addition, CD163 may play a role in host defense by regulating the release of cytokines by macrophages [27]. Some investigations have also identified CD163 as a macrophage receptor for bacteria and suggested that during bacterial infection, CD163 on resident tissue macrophages acts as an innate immune sensor and inducer of local inflammation [28]. However, the high levels of CD163 in liver failure may represent an anti-inflammatory imbalance, especially in patients with poor disease outcomes. This theory is supported by a recent report that describes a significant reduction in the expression of monocytic HLA-DR in patients with acute liver failure and importantly, a correlation between monocytic deactivation and poor survival [6]. Monocytes with high CD163 expression show an impaired ability to present antigens and produce TNF-α [6,29], and may represent a possible defect that is directly linked to the development of recurrent bacterial and opportunistic infections [30].

CD163 protein expression is closely regulated and associated with the innate immune response to infection. Consistent with a role in the late inflammatory response, the expression of CD163 is up-regulated by glucocorticoids, IL-10, IL-6 and PGE2 but down-regulated by lipopolysaccharides (LPS), IFN-γ, TNF-α and TGF-β [14,31-33]. PGE2 belongs to a family of short-lived chemical paracrine messengers and has been studied the past 20 years for its immunomodulatory properties [34]. Several studies have clearly indicated a positive effect of PGE2 on up-regulation of CD163 on monocytes [14]. Similarly, we found that PGE2 is sufficient to stimulate monocytes to express CD163. However, we were unable to fully abolish CD163 expression with NS-398, and furthermore, normal skin fibroblasts showing no secretion of PGE2 also partially affected CD163 expression, suggesting that PGE2-independent mechanisms may also be involved in the activation of monocytes in monocyte/LMF cocultures. Further studies are required to confirm those pathways.

LMFs originate principally from activated hepatic stellate cells. However, in fibrotic disease, subpopulations arise from other sources, such as bone-marrow precursors [35-37]. It is believed that myofibroblasts isolated from tissues express imprinted phenotypes that are stable in culture [38]. Therefore, the behavior of these cells in vitro is likely to reflect their function in vivo [39]. We studied differentiated LMFs isolated directly from different diseased human livers. The myofibroblasts we isolated were positive for α-SMA and FAP, and characteristic markers of epithelial, endothelial or hematopoietic cells, such as CD31, CD45 and CD34, were absent. There were no consistent differences that characterized LMFs isolated from different patients with hepatitis B induced liver failure, and all LMFs expressed similar types of markers and could secrete similar levels of PGE2 (data not shown). Consistent with our results, other investigators have recently reported that LMF preparations from different diseased livers secreted similar patterns of proinflammatory cytokines and chemokines [18].

Our study has limitations. First, except for the role of myofibroblast-derived PGE2 that increases CD163 expression on monocytes/macrophages, the data are descriptive or correlative, lacking mechanistic or functional studies. It remains unclear what PGE2’s role relative to other potent CD163 inducers (e.g., IL-10 or corticosteroids) is and what the signal transduction is. Nevertheless, our study is of hypothesis-generating value for the design of future in vitro studies. Second, the 20 patients from whom serum was studied and the 4 liver tissues of subjects with hepatitis B induced liver failure are not representative for all phases of hepatitis B and these numbers are small. Consistent with our results, other investigators have reported the up-regulation of CD163 in patients with liver failure [5]. Nonetheless, since there were no consistent differences that characterized LMFs isolated from different patients, we believe that this phenomenon is not peculiar to a few patients and as seems more likely applicable to most patients with hepatitis B induced liver failure. Despite these shortcomings, we believe our analysis shows the role of LMFs in the regulation of CD163 expression on monocytes during the progression of liver injury. Larger studies are necessary to fully address this important question, as well as to assess the impact of other possible determinants of CD163 expression.

## Conclusions

Our results provide important new insights into the role of LMFs in the regulation of CD163 expression on monocytes. PGE2 derived from LMFs can trigger increased expression of CD163 on recruited monocytes in liver and thereby induce the monocytes to shift towards an immunosuppressive or anti-inflammatory state. Therefore, studying the mechanisms that enable LMFs to selectively modulate the functional activities of monocytes may provide a novel strategy for the treatment of liver failure.

## Abbreviations

Abs: Antibodies; APC: Antigen-presenting cell; α-SMA: Alpha-smooth muscle actin; BSA: Bovine serum albumin; COX: Cyclooxygenase; ELISA: Enzyme-linked immunosorbent assay; FAP: Fibroblast activation protein; FCS: Fetal calf serum; HBV: Hepatitis B virus; HSCs: Hepatic stellate cells; IgG: Immunoglobulin; LMFs: Liver myofibroblasts; LPS: Lipopolysaccharides; PBS: Phosphate-buffered saline; PBMCs: Peripheral blood mononuclear cells; PGE2: Prostaglandin E2; SRCR: Cavenger-receptor cysteine-rich.

## Competing interests

All authors declare that they have no competing interests.

## Authors’ contributions

MZ and YY performed cell isolations, cell-coculture studies, flow cytometry analyzes and western blotting, created figures and contributed to the design of the study and the writing of the manuscript. FW and JZ performed cell isolations and flow cytometry studies. YZ, QZ and YG performed flow cytometry, ELISA and assisted in liver myofibroblasts evaluation and in manuscript preparation. CX, ZH and QT contributed to the design of the study, experimental design and the writing of the manuscript. YC and ZG (the corresponding authors) designed and coordinated the study, contributed to experimental setup, data analysis and interpretation, and drafted and edited the manuscript. All authors read and approved the final manuscript.

## Supplementary Material

Additional file 1: Table S1 Basic clinical characteristics of the patients. Click here for file
