# Supplementary material for: Liver myofibroblasts up-regulate monocyte CD163 expression via PGE2 during hepatitis B induced liver failure
Source: J Transl Med. 2014 Mar 6;12:60. doi: 10.1186/1479-5876-12-60 (PMC4015181; doi:10.1186/1479-5876-12-60)
Supplement: Additional file 1: Table S1 — Basic clinical characteristics of the patients. [file 1479-5876-12-60-S1.doc]

Supplementary Table 1. Basic clinical characteristics of the patients

| **Case** | **Age**  **(years)** | **Sex** | **ALT**  **(U/L)** | **TBIL**  **(umol/L)** | **HBsAg** | **HBeAg** | **Cirrosis** | **Liver tissue** | **MELD score** |
| --- | --- | --- | --- | --- | --- | --- | --- | --- | --- |
| 1 | 23 | Male | 186 | 447 | Positive | Positive | No | No | 25 |
| 2 | 41 | Male | 11 | 492 | Positive | Positive | No | No | 44 |
| 3 | 29 | Male | 501 | 276 | Positive | Negative | No | No | 26 |
| 4 | 31 | Male | 43 | 742 | Positive | Positive | No | Yes | 36 |
| 5 | 33 | Male | 63 | 658 | Positive | Negative | Yes | No | 28 |
| 6 | 36 | Male | 371 | 536 | Positive | Negative | Yes | No | 31 |
| 7 | 32 | Male | 741 | 472 | Positive | Negative | No | No | 33 |
| 8 | 47 | Male | 107 | 766 | Positive | Positive | Yes | No | 35 |
| 9 | 53 | Male | 58 | 570 | Positive | Positive | Yes | Yes | 26 |
| 10 | 49 | Male | 51 | 706 | Positive | Negative | Yes | No | 29 |
| 11 | 61 | Male | 49 | 597 | Positive | Negative | Yes | No | 30 |
| 12 | 47 | Male | 108 | 788 | Positive | Negative | No | No | 38 |
| 13 | 24 | Female | 47 | 654 | Positive | Positive | No | No | 25 |
| 14 | 35 | Male | 110 | 722 | Positive | Positive | No | No | 27 |
| 15 | 61 | Male | 104 | 770 | Positive | Positive | No | Yes | 37 |
| 16 | 43 | Male | 260 | 610 | Positive | Negative | No | No | 29 |
| 17 | 55 | Male | 204 | 177 | Positive | Positive | Yes | No | 24 |
| 18 | 58 | Male | 106 | 258 | Positive | Positive | Yes | Yes | 32 |
| 19 | 43 | Male | 63 | 174 | Positive | Negative | No | No | 27 |
| 20 | 68 | Female | 112 | 634 | Positive | Negative | Yes | No | 33 |

Abbreviations: ALT, alanine aminotransferase; TBIL, total bilirubin; HBsAg, hepatitis B surface antigen; HBeAg, hepatitis B e antigen; MELD, model for end-stage liver disease
